# Acupuncture for chronic urticaria: a systematic review and meta-analysis with trial sequential analysis

**DOI:** 10.3389/fneur.2025.1650418

**Published:** 2026-01-21

**Authors:** Ke-Xin Wu, Yan Chen, Peng Tang, Jin Yao, Xin-Yue Zhang, Qiong-Nan Bao, Ya-Qin Li, Zi-Wen Chen, Wan-Qi Zhong, Man-Ze Xia, Zheng-Hong Chen, Zi-Han Yin, Fan-Rong Liang

**Affiliations:** 1 Acupuncture and Tuina School, Chengdu University of Traditional Chinese Medicine, Chengdu, China; 2 Sichuan Provincial Acupuncture Clinical Research Center, Chengdu, China; 3 Wenjiang District Chinese Medicine Hospital, Chengdu, China; 4 The First People's Hospital of Yunnan Province, Kunming, China; 5 The Fourth People's Hospital of Chengdu, Chengdu, China; 6 University of Xizang Medicine, Lhasa, China

**Keywords:** chronic urticaria, acupuncture, meta-analysis, trial sequential analysis, systematic review

## Abstract

**Background and purpose:**

Chronic urticaria (CU) manifests as recurrent skin wheals and itching, considerably impacting patient quality of life. This study aimed to evaluate the efficacy and safety of acupuncture treatment for CU using meta-analysis and trial sequential analysis (TSA), providing a basis for clinical decision-making.

**Methods:**

A systematic search was performed across six English databases, four Chinese databases, and additional resources up to 30 September 2025. Randomized controlled trials comparing acupuncture with Western medicine (WM), sham acupuncture (SA), and waitlist control (WC) were included. The revised Cochrane Collaboration Risk of Bias tool was used to assess methodological quality. Review Manager (version 5.4) and STATA (version 17) were used for statistical analysis and complex modeling, respectively. The Grading of Recommendations Assessment, Development, and Evaluation (GRADE) system was used to evaluate the evidence level, and TSA was used to estimate the required sample size and evaluate the stability of the study conclusions. Subgroup analyses were performed based on acupuncture methods and control methods.

**Results:**

The analysis included 18 studies involving 1,829 patients. Acupuncture demonstrated a significant advantage over SA and WC in reducing the Weekly Urticaria Activity Score (UAS7), while no significant difference was observed compared to WM. For secondary outcomes, acupuncture demonstrated a significant advantage over WM, SA, and WC in terms of the Dermatology Life Quality Index (DLQI). TSA's findings indicated that the evidence of reducing UAS7 and DLQI scores with acupuncture for patients with urticaria was conclusive. Meanwhile, the difference between acupuncture and WM regarding serum IgE levels was statistically non-significant. TSA showed that the evidence of improving IgE levels is inconclusive. The incidence of adverse effects associated with acupuncture treatment, including ecchymosis and pain, was higher than that in SA and WC.

**Conclusion:**

Acupuncture is a potential therapeutic intervention for CU, capable of reducing the frequency of urticarial episodes and significantly improving patient symptoms and quality of life.

**Systematic review registration:**

https://www.crd.york.ac.uk/prospero/, identifier: CRD42023480490.

## 1 Introduction

Chronic urticaria (CU) is caused by many factors resulting in temporary inflammatory congestion of the skin, mucous membranes, blood vessels, and tissue edema (1). It is characterized by daily or almost daily hives, which may be accompanied by pruritus, with or without angioedema, lasting at least 6 weeks (2). The global incidence of CU ranges between 0.5% and 1.0%, with a lifetime prevalence as high as 1.8% and women exhibiting about double the prevalence in men (3, 4). CU causes anxiety, depression, irritability, and social dysfunction, which significantly affects the quality of life and emotional health of those affected. Additionally, it incurs a substantial economic burden, with the total average annual direct expenses per patient ranging from $907.1 to 2,984.2 (5, 6). Current guidelines recommend second-generation antihistamines as the first-line treatment for CU; however, they do not completely alleviate symptoms (7, 8). Although the monoclonal antibody omalizumab demonstrates excellent efficacy, its relatively high cost limits its use (9, 10). Therefore, other safer and more effective treatment methods are required.

Acupuncture, a complementary and alternative medical technique, has long been used to treat pruritus. Acupuncture combined with other acupuncture therapies is as effective as Western medicine (WM) in treating CU (11–13)]. Acupuncture possesses significant benefits in managing patient symptoms, boosting therapeutic effectiveness, and improving cure rates (14, 15). Furthermore, acupuncture can reduce symptoms by influencing immune cell and inflammatory mediator release (16–19). Recently, Hui Zheng discovered that acupuncture improved the severity of itching and wheals more significantly than sham acupuncture (SA) and the waitlist control (WC) (20).

Previous systematic reviews have been conducted, and small sample sizes in randomized controlled trials (RCTs) may introduce biases and increase the risk of false-positive results. Currently, no studies have estimated the sample size for the included research. Trial sequential analysis (TSA) can determine the required information size (RIS) and assess the futility boundary, thereby reducing the risk of false-positive results due to inaccurate meta-analyses and repeated significance tests (21, 22). Furthermore, the diversity of outcome measures and the quality of evidence from RCTs raise concerns about the efficacy of acupuncture treatment for CU and the reliability of the existing evidence. Consequently, using the UAS7 as the primary outcome measure, this study evaluated the efficacy and safety of acupuncture treatment for CU via meta-analysis and TSA, providing evidence for clinical CU treatment.

## 2 Materials and methods

### 2.1 Design and registration

The meta-analysis was designed and conducted following the Preferred Reporting Items for Systematic Reviews and Meta-Analyses (PRISMA) guidelines for evaluating the efficacy and safety of acupuncture in treating CU (23, 24). The meta-analysis is registered in the PROSPERO database (registration number: CRD42023480490).

### 2.2 Search strategy

We searched the following databases from their inception to September 2025: Embase, PubMed, Cochrane Library, Web of Science, Allied and Complementary Medicine, Cumulative Index to Nursing and Allied Health Literature, China National Knowledge Infrastructure, Wanfang, VIP Chinese Medical Journal Database, and China BioMedical Literature database. The publication language was limited to English and Chinese. The following search terms were used: (1) disease (CU), (2) intervention (acupuncture), and (3) type of study (RCT). Furthermore, we conducted forward and backward citation tracking based on the references included in the studies (Supplementary File S1).

### 2.3 Eligibility criteria

RCTs satisfying the following criteria were included: (1) Population: patients diagnosed with CU (25), regardless of gender, age, race, or region; (2) intervention: acupuncture, including manual acupuncture (MA), electroacupuncture, warm acupuncture, and fire acupuncture, with (or without) the same intervention measures as the control group. Acupuncture point selection, reinforcing and draining techniques, retention time, choice of needles, and electric acupuncture parameters were unrestricted; (3) comparison, including WM, SA, and WC; (4) outcomes: the primary outcome was the change in the UAS7. Secondary outcomes included changes in the Dermatology Life Quality Index (DLQI) and serum IgE levels before and after treatment; as well as the incidence of adverse events. (5) study design: RCT. The exclusion criteria were as follows: (1) unavailability of full text or insufficient data for analysis after attempting to contact the corresponding authors; (2) publication type was a review, case report, conference abstract, expert experience, letter review or animal study.

### 2.4 Study selection

The output from all searches was imported into the Endnote (version X9) software for management. After removing duplicates, two reviewers independently assessed the relevance of the titles, keywords, and abstracts of the retrieved articles using the inclusion and exclusion criteria. They further screened the full texts of potentially eligible RCTs. Eligibility for the remaining studies was determined after reading the full texts. Any disagreements were resolved by discussion or consultation with a third author.

### 2.5 Data extraction

Two researchers extracted the data independently using a pre-designed extraction form. Any disagreements were resolved by the corresponding authors. The data extracted included the following: (1) basic characteristics (first author, year of publication, and country); (2) general information (sample size, study design, and distribution ratio); (3) participants (age, gender, and course of disease); (4) intervention characteristics (name of intervention or control, dose and frequency of treatment, and treatment duration); (5) outcomes [primary outcome, secondary outcomes and adverse events (AEs)]. For missing data, we contacted the authors by email to request the original data. When data from the author was unavailable, we estimated the data using the availability coefficient (26).

### 2.6 Assessment of RoB

Two reviewers independently assessed the methodological quality of the selected RCTs using the Risk of Bias (RoB, version 2.0) assessment tool from the Cochrane Collaboration (27). The tool addresses five types of biases: (1) the randomization process, (2) deviation from the intended intervention, (3) missing outcome data, (4) result measurement, and (5) selection of reported results. The included trials were categorized as having “a low RoB,” “some concerns,” or “a high RoB.” A third reviewer was consulted during the final decision-making process.

### 2.7 Statistical analysis

Data were analyzed using Review Manager (version 5.4) and STATA (version 17) software. Continuous variables were analyzed using mean difference (MD) and 95% confidence interval (CI). For binary variables, relative risk (RR) was used as the effect scale at 95% CI. Statistical significance was set at *P* < 0.05 (two-tailed). The random-effects model was applied for all meta-analyses to account for anticipated clinical heterogeneity among studies. For multi-arm trials, the sample size of the acupuncture group was split evenly between independent comparisons (e.g., MA vs. SA and MA vs. WC) to avoid double-counting, in accordance with the Cochrane Handbook (28). Heterogeneity was quantified using the *I*^2^ statistic, with *I*^2^ ≥ 50% considered substantial. Subgroup or sensitivity analyses were conducted to investigate heterogeneity sources. When inter-study heterogeneity was too high, a descriptive analysis was conducted. Publication bias was analyzed using Egger's test, with *P* > 0.05 indicating a lower risk of publication bias. AEs were analyzed for safety using risk ratios (RR), with the occurrence rate calculated as (Total AEs/Total participants) × 100%.

### 2.8 Sequential analysis of trials

A sequential analysis of outcome measures was performed using TSA (version 0.9.5.10) Beta software from the Copenhagen Clinical Trial Center. Traditional meta-analysis does not estimate sample size. In meta-analysis, TSA adjusts for random error and estimates the sample size required to ensure the statistical reliability of the data (Supplementary File S3).

### 2.9 Grading the evidence

The Grading of Recommendations Assessment, Development, and Evaluation (GRADE) (29, 30) system was used to evaluate the quality of evidence, which was rated as “high,” “moderate,” “low,” or “very low.” Two reviewers conducted the assessment; in cases of disagreement, a decision was made after consulting a third author.

## 3 Results

### 3.1 Study description

#### 3.1.1 Literature search

**Figure 1:**
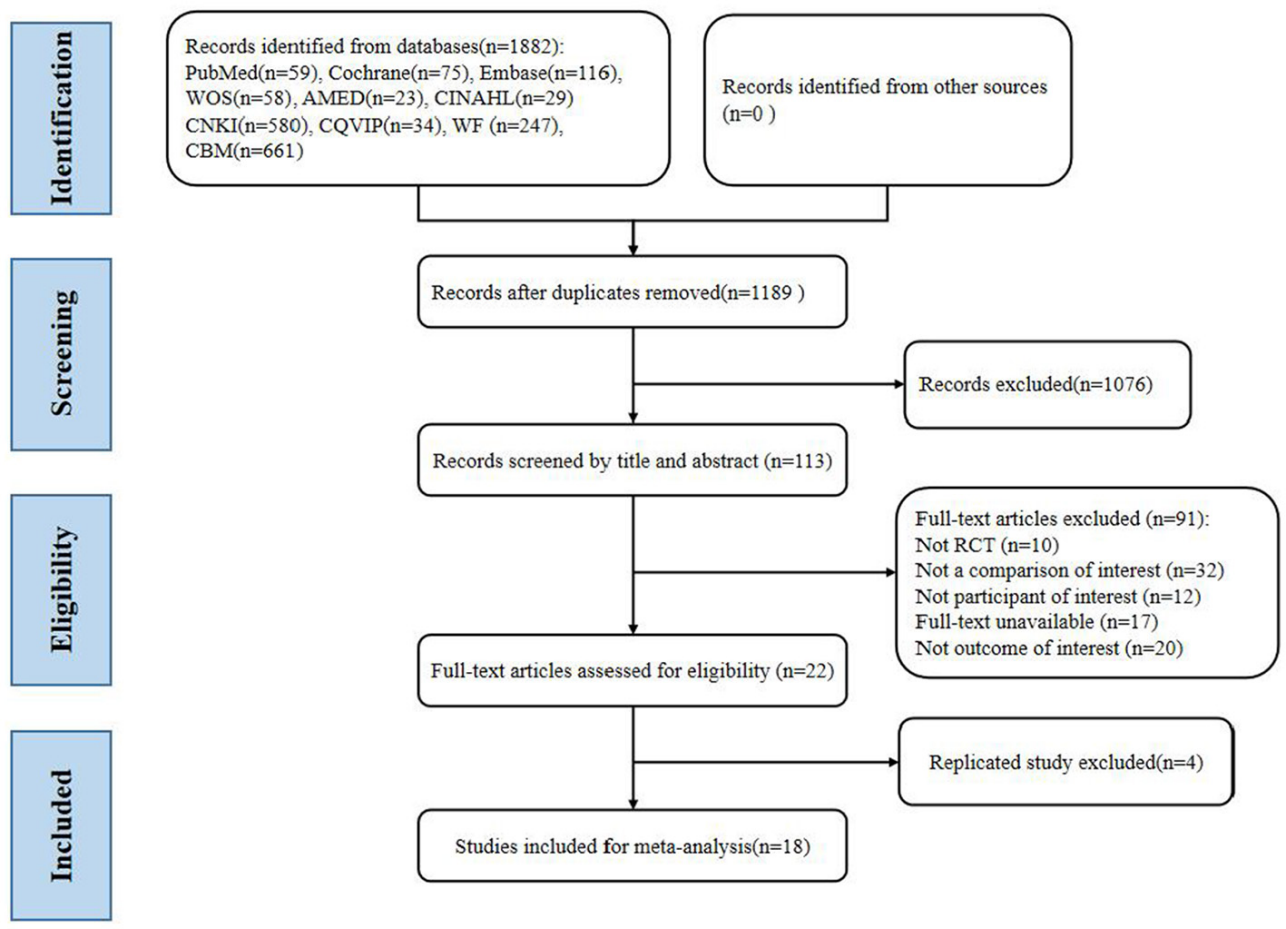
PRISMA flow diagram showing four phases of the study.

A total of 1,882 relevant articles were retrieved from eight databases (Figure 1). After removing duplicate publications, reviewing titles and abstracts, and doing a full-text review, we included 22 articles that met the criteria for this study, 4 of which were from the same clinical study. Thus, 18 studies were included (20, 31–47), with 4 3-arm studies divided into 8 trials for comparison (20, 34, 36, 37).

#### 3.1.2 Characteristics of the included RCTs

The 18 studies originated from China, with publication years ranging from 2009 to 2024, 12 of which were published in the last 5 years. The 18 studies included 1,829 patients, with sample sizes ranging from 60 to 330 cases. The ages of the patients ranged from 18 to 65 years, and the disease duration spanned from 1.5 to 250.8 months. The experimental group received acupuncture, whereas the control group received oral medication (loratadine/cetirizine hydrochloride), SA, or WC. A total of 11 independent comparisons from 7 studies (20, 32–34, 36, 37, 47) reported data on the UAS7, 15 comparisons from 11 studies (20, 31, 33–37, 39, 43, 44, 46) reported the DLQI, and 7 studies (38–42, 45, 46) reported the effect of acupuncture on serum IgE levels. This discrepancy between the number of studies and comparisons arises because the 4 three-arm studies (20, 34, 36, 37) each contributed two independent comparisons to the meta-analyses. Six studies reported follow-up information. The characteristics of the included literature are presented in Table 1.

**Table 1:** Characteristics of the included RCTs.

**Study**	**Sample size**	**Allocation ratio**	**Male: female**	**Course of disease**	**Age (year)**	**Interventions**	**Treatment frequency/period**	**Follow-up time**	**Outcomes**	**Lost to follow-up**
Nan Ge 2024	62	31:31	A.10:21 B.11:20	A.12.81 ± 4.01 m B.13.55 ± 4.37 m	A.37.48 ± 9.51 B.37.48 ± 9.51	MA vs. WM (Cetirizine hydrochloride)	A. 5 times/w, 4 w B. 10 mg, q d, 4 w	–	1.Urticaria Symptom Scores; 2. DLQI; 3. IgE	–
Lu Cao 2024	60	31:29	A.8:23 B.8:21	A.50.00 ± 42.22 m B.31.00 ± 35.56 m	A.31.00 ± 16.30 B.40.00 ± 21.48	MA vs. WM (Desloratadine citrate disodium)	A. 5 times/2 w; 3 times/2 w, 4 w B. 8.8 mg, q d,4 w	4 w	1. UAS7; 2. VAS; 3.UCT; 4.Weekly angioedema activity score	A.0 B.0
Yunzhou Shi 2023	80	41:39	A.14:27 B.11:28	A.38.4 (1.5–336) m B. 39.5 (1.50–149) m	A:38.2 (19–69) B:39.7 (21–66)	MA vs. SA	A. 5 times/w, 2 w B. 5 times/w, 2 w	2 w	1. UAS7; 2.VAS; 3. DLQI; 4. HAMD; 5. HAMA	A.1 (1.3%) B.4 (5%)
Wenna Shu 2023	62	31:31	A.14:17 B.17:14	A.11.2 ± 9.7 y B.8.2 ± 7.3 y	A.38.6 ± 15.2 B.37.2 ± 14.3	MA (Warm moxibustion) vs. WM (Levocetirizine hydrochloride)	A. 5 times/w, 3 w B. 5 mg q d, 3 w	–	1. CSSoCU; 2. SSRI; 3. DLQI	A.0 B.0
Shengyuan Qu 2023	74	37:37	A.11:21 B.11:17	A.40.50 ± 74.07 m B.48.00 ± 58.89 m	A.45.01 ± 11.76 B.43.57 ± 11.66	MA vs. SA	A. 3 times/w, 4 w B. 3 times/w, 4 w	4 w	1. UAS7; 2. Cu-Qol; 3. UCT; 4. SAS; 5. SDS; 6. ISI	A.5 B.9
Hui Zheng 2023	330	110:110:110	A.25:85 B.31:79 C.33:77	A.4.2 (3.2–5.2) y B.4.3 (3.1–5.5) y C.4.0 (3.0–5.0) y	A.38.4 (36.0–40.9) B.39.2 (36.7–41.7) C.40.4 (38–42.8)	MA vs. SA vs. WC	A. 10 times/2 w; 6 times/2 w B. 10 times/2 w; 6 times/2 w	4 w	1. UAS7; 2. itch severity scores; 3. self-rated improvement; 4. DLQI; 5. lower rate of antihistamine use; 6. HAMA;7. HAMD;8. PSQI	A.7 B.15 C.12
Zhongxun Wang 2021	70	35:35	–	–	–	MA vs. WM (Levocetirizine hydrochloride)	A. 3 times/w, 4 w B. 1 pill, q d, 1 m	–	1. UAS; 2. DLQI; 3. HAMA; 4. SSRI; 5. Effective rate	A.1 B.4
Xianjun Xiao 2021	202	67:67:68	A.15:52 B.18:49 C.21:47	A.30.33 ± 40.74 B.32.67 ± 40 C.30.92 ± 40.56	A.36.33 ± 14.82 B.35.33 ± 14.82 C.38.67 ± 17.78	MA vs. SA vs. WC	A. 10 times/2 w; 6 times/2 w, 4 w B. 10 times/2 w; 6 times/2 w, 4 w	4 w	1. USA7; 2.VAS; 3. PSQI; 4. DLQI; 5. HAMA; 6. HAMD	A.3 B.8 C.2
Lu Wang 2021	75	24:26:25	A.6:18 B.7:18 C.7:16	A.3.04 ± 3.22 y B.3.28 ± 3.86 y C.2.04 ± 1.77 y	A.31.83 ± 8.26 B.32.36 ± 10.78 C.37.91 ± 13.40	MA vs. SA vs. WC	A. 10 times/2 w; 6 times/2 w B. 10 times/2 w; 6 times/2 w	4 w	1. UAS7; 2. DLQI; 3. HAMD; 4. PSQI	A.0 B.1 C.2
Leixiao Zhang 2021	60	22:19:19	A.4:14 B.5:13 C.5:12	A.83.18 ± 44.40 m B.85.11 ± 48.18 m C.84.89 ± 53.30 m	A.41.05 ± 12.50 B.42.05 ± 13.31 C.41.16 ± 12.74	MA vs. SA vs. WC	A. 10 times/2 w; 6 times/2 w B. 10 times/2 w; 6 times/2 w	–	1. UAS7; 2.VAS; 3.DLQI; 4.HAMA, HAMD; 5. PSQI	A.4 B.1 C.2
Wenyin Wu 2020	106	53:53	A.25:28 B.27:26	A.17 ± 5 (9–61) m B.18 ± 6 (10–60) m	A.42 ± 5 (22–65) B.42 ± 6 (21–64)	MA vs. WM (Loratadine)	A. 5 times/w, 8 w B. 10 mg, q d, 8 w	–	1. DLQI; 2. IL-4, IgE, IFN; 3. Effective rate	–
Haiyan Gu 2020	90	45:45	A.18:27 B.17:28	A.22.54 ± 6.57 (16–32) m B.23.21 ± 7.70 (17–31) m	A.40.76 ± 8.86 (36–59) B.41.79 ± 7.49 (37–58)	MA vs. WM (Loratadine)	A. 5 times/w, 6 w B. 10 mg, q d, 6 w	–	1. CSSoCU; 2.IL-4, IgE, IFN; 3. Effective rate	A.1 B.1
Libin Gu A 2019	158	78:78	A.50:28 B.48:30	A.3.41 ± 1.09 y B.3.49 ± 1.18 y	A.37.28 ± 11.91 (19–72) B.37.14 ± 11.37 (18–29)	MA vs. WM (Loratadine)	A.–, 8 w B. 10 mg, q d, 8 w	–	1. CSSoCU; 2.IL-4, IgE, IFN; 3. Effective rate	–
Libin Gu B 2019	120	60:60	A.26:34 B.28:32	A.16.35 ± 12.08 (8–60) m B.17.01 ± 13.11 (9–65) m	A.40 ± 12 (18–64) B.41 ± 12 (20–60)	MA vs. WM (Loratadine)	A. 5 times/w, 6 w B. 10 mg, q d,6 w	2 w	1. CSSoCU; 2.IL-4, IgE, IFN; 3. Effective rate	–
Liangnan Zhang 2019	90	45:45	A.18:27 B.17:28	A.22.54 ± 6.57 (16–32) m B.23.21 ± 7.70 (17–31) m	A.40.76 ± 8.86 (36–59) B.41.79 ± 7.49 (37–58)	MA vs. WM (Loratadine)	A. 5 times/w, 6 w B. 10 mg, q d, 6 w	1 m	1. CSSoCU; 2.IL-4, IgE, IFN; 3. Effective rate	A.1 B.1
Anqi Dai 2014	60	30:30	A.14:16 B.15:15	A.11.07 ± 4.59 m B.13.00 ± 5.35 m	A.44.00 ± 14.49 B.41.70 ± 11.93	MA (Warm moxibustion) vs. WM (Levocetirizine hydrochloride)	A. 3 times/w, 3 w B. 10 mg, q d, 6 w	–	1. CSSoCU; 2. DIQL	A.0 B.0
Jing Liang 2013	70	35:35	A.17:18 B.15:20	–	–	MA vs. WM (Loratadine)	A. 2 times/w, 8 w B. 10 mg, q d, 8 w	8 w	1. UAS; 2. DLQI; 3. Effective rate	A.0 B.0
Hong Gao 2009	60	30:30	A.13:17 B.11:19	A.15.87 ± 13.07 m B.16.17 ± 14.40 m	A.35.6 ± 13.8 (18–60) B.37.5 ± 12.9 (19–60)	MA vs. WM (Levocetirizine hydrochloride)	A. 5 times/w, 2 w B. 5 mg, q d, 6 w	10 w	1. IgE; 2.C3; 3. CSSOCU	–

MA, manual acupuncture; SA, sham manual acupuncture; UAS7, weekly urticaria activity score; DLQI, dermatology life quality index scores; VAS, visual analog scale; HAMD, Hamilton depression scale; HAMA, Hamilton anxiety scale; SAS, self-rating anxiety scale; SDS, self-rating depression scale; ISI, insomnia severity index; UCT, urticaria control test; IL-10, interleukin-10; WM, western medicine; CSSoCU, clinical symptom and sign score of chronic urticarial.

### 3.2 Quality assessment

**Figure 2:**
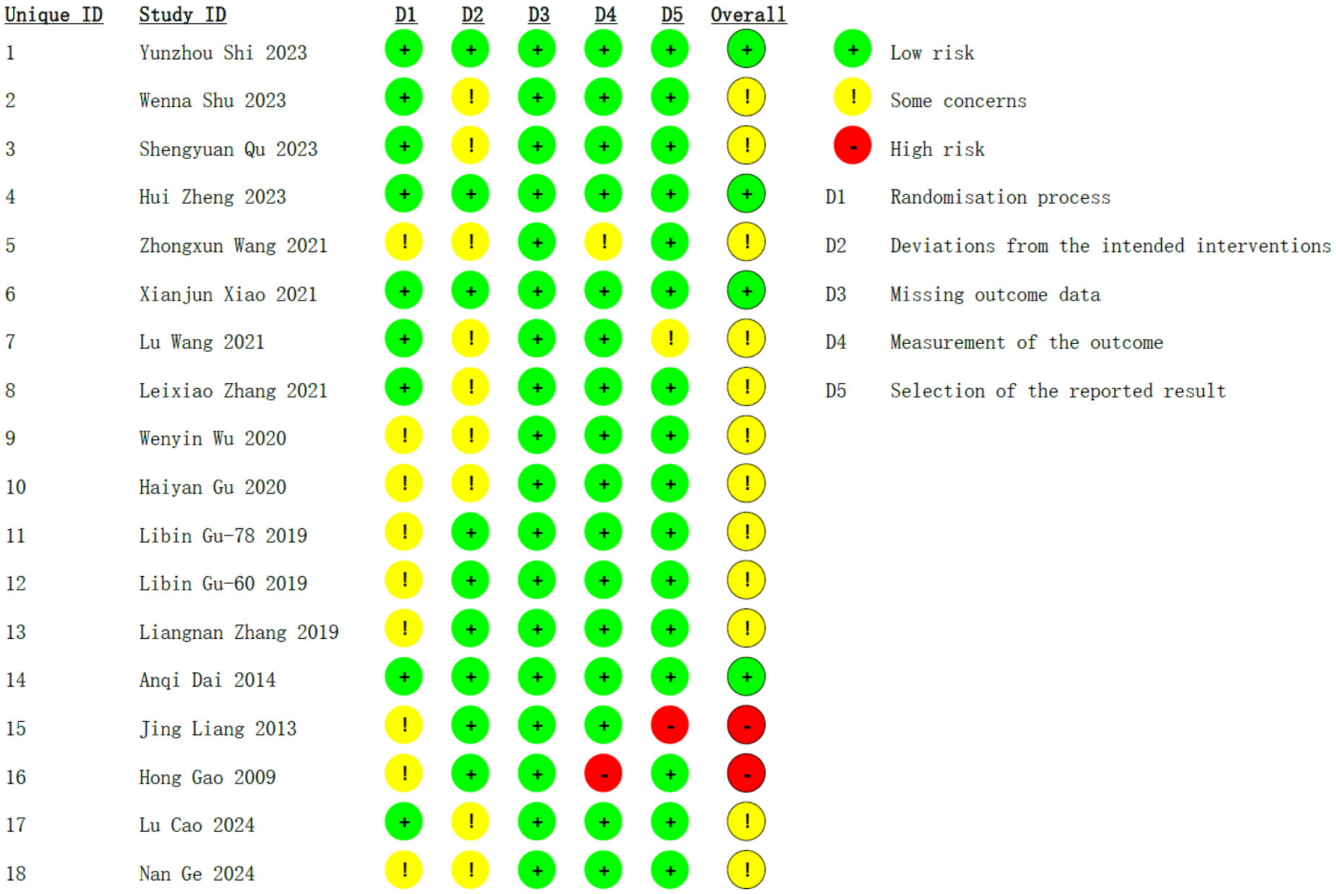
Risk of bias assessment.

According to the Cochrane Handbook and the RoB tool (48), in the section on the randomization process, nine trials were categorized as low risk, and the rest of the trials had certain concerns. For allocation concealment, nine studies exhibited an unclear RoB. In terms of blinding of participants and personnel, nine studies raised some concerns, whereas all studies were judged to have a low risk of bias for blinding of outcome assessment. One study exhibited an unclear risk of bias for the integrity of data outcomes, and one study was at high risk. In the selection of the reported results, one study demonstrated an unclear risk and one was assessed as high risk. Overall, 4 studies were at low risk, 2 were at high risk, and the remaining 12 were at moderate risk (Figure 2).

### 3.3 Effects of intervention

#### 3.3.1 Change in the UAS7

**Figure 3:**
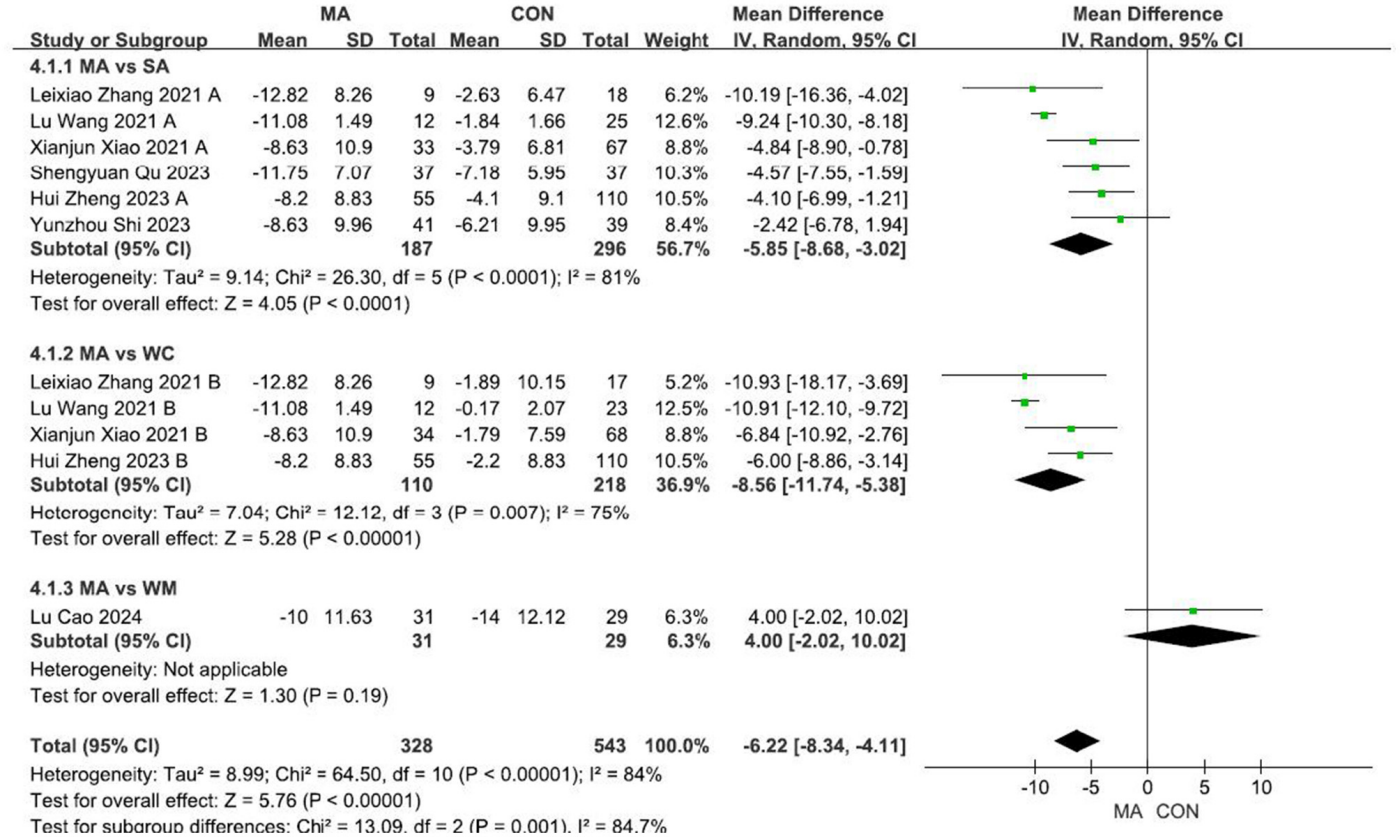
Forest plot comparing the UAS7 between the acupuncture and control groups.

A total of 11 independent comparisons reported the UAS7 before and after treatment, with a total sample size of 871 cases. Due to the high heterogeneity of the studies, a random-effects model was used for analysis. The acupuncture group exhibited a significantly reduced UAS7 compared with the control group [MD = −6.22 (−8.34, −4.11); *P* < 0.00001; Figure 3]. Six RCTs reported follow-up results, with the acupuncture group outperforming the non-acupuncture groups [MD = −5.61 (−8.30, −2.92); *P* < 0.0001; Supplementary Figure S1].

**Figure 4:**
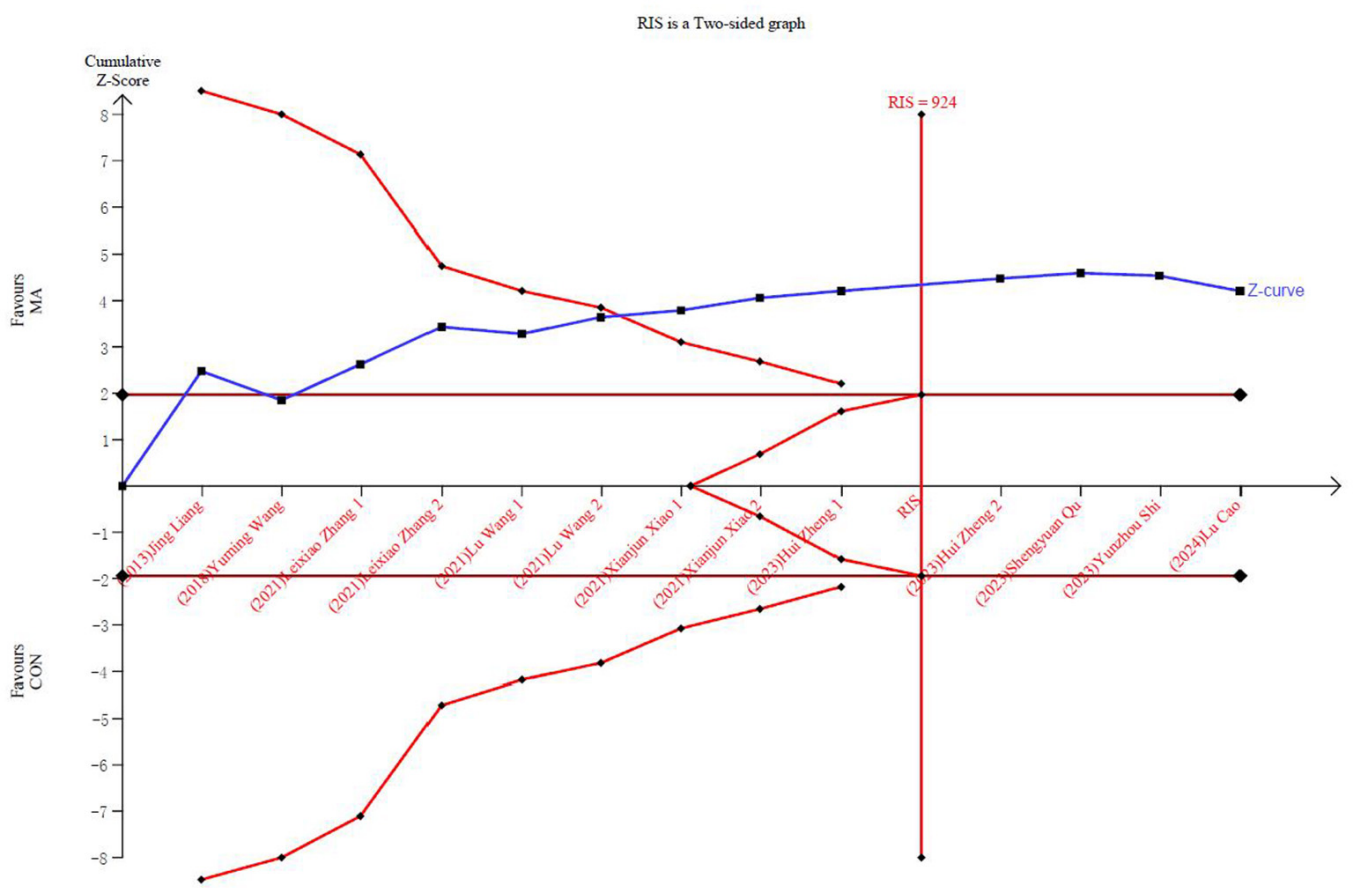
Trial sequential analysis of the UAS7 adjusted boundaries.

**Figure 5:**
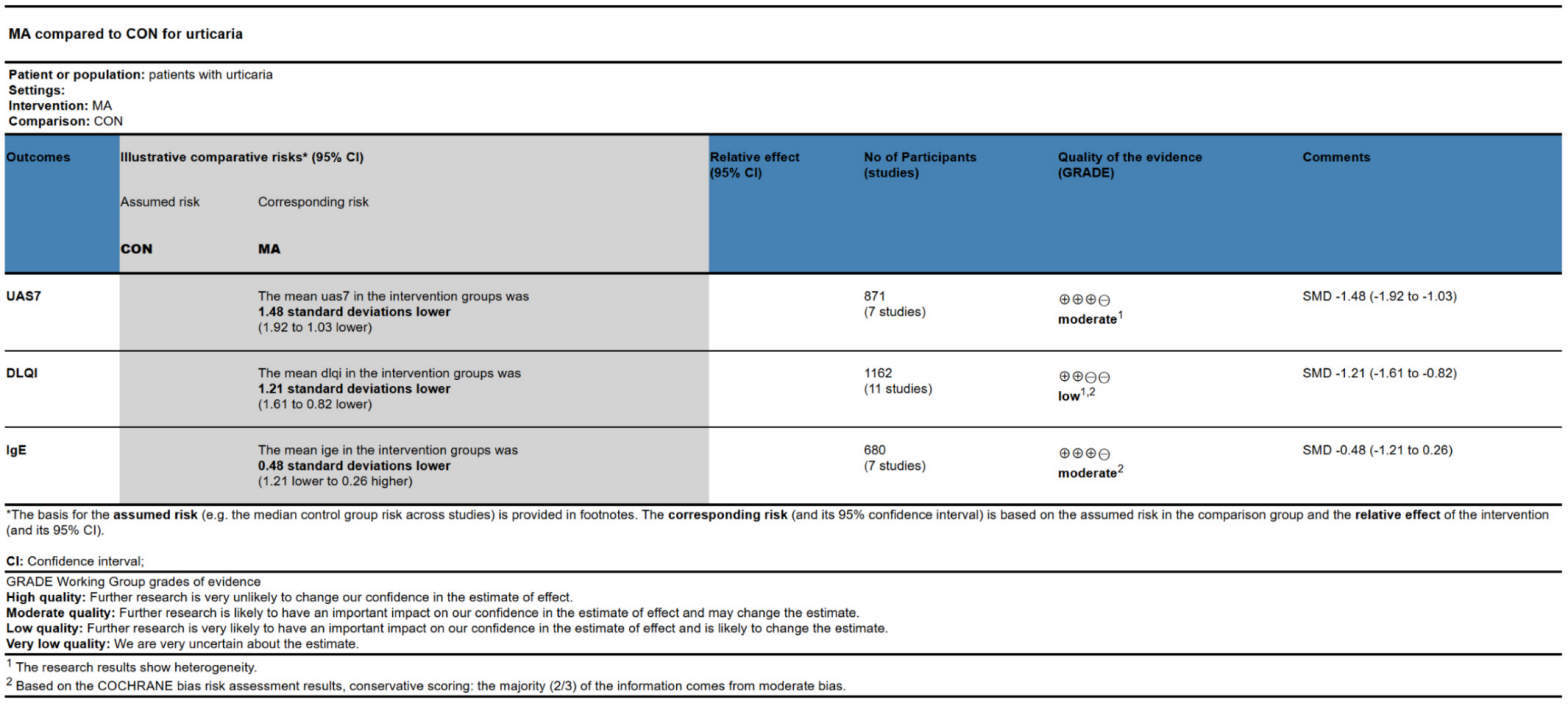
Quality assessment of reported results using the GRADE approach.

In the TSA, the Z-curve crossed the conventional test boundary and exceeded the effectiveness and RIS boundaries, indicating that the meta-analysis results are robust (Figure 4). Egger's test (Supplementary Figure S2) for small-study effects based on regression suggested a potential publication bias (*P* = 0.005). Using the GRADE tool to rate the quality of evidence for the decrease in the UAS7, we determined that it was moderate (Figure 5).

Given the high heterogeneity observed, we conducted subgroup analyses based on different control measures. We included six studies comparing acupuncture with SA (Figure 3). Acupuncture outperformed SA in reducing the UAS7 [MD = −5.85 (−8.68, −3.02); *P* < 0.0001]. Four studies met the inclusion criteria for the comparison of acupuncture and the WC, and acupuncture outperformed the WC in reducing the UAS7 [MD = −8.56 (−11.74, −5.38); *P* < 0.00001]. One study compared MA with WM, and found no significant difference (*P* = 0.19). There were no significant differences between the subgroups.

#### 3.3.2 Changes in the DLQI

A total of 15 independent comparisons reported the DLQI before and after treatment, with an overall sample size of 1,162 cases. Due to the high heterogeneity of the studies, a random-effects model was used for analysis. The results revealed that acupuncture reduced the DLQI, indicating an improvement in quality of life for patients with CU, with a significantly better therapeutic effect than the controls [MD = −3.92 (−4.97, −2.87); *P* < 0.00001; Supplementary Figures S3, S4]. Four RCTs reported follow-up results, with acupuncture outperforming the controls [MD = −3.34 (−4.25, −2.44); *P* < 0.00001; Supplementary Figure S5]. In the TSA, the Z-curve crossed the conventional test boundary and exceeded the effectiveness and RIS boundaries, indicating that the meta-analysis results are robust (Supplementary Figure S6). Egger's test suggested a potential publication bias (*P* < 0.001; Supplementary Figure S7). Using the GRADE tool, the quality of evidence for changes in the DLQI was rated low (Figure 5).

Due to the high heterogeneity observed between the control groups (SA, WM, and WC), we employed subgroup analysis (Supplementary Figure S3). We included five studies comparing acupuncture with SA. Acupuncture outperformed SA in reducing the DLQI [MD = −3.60 (−5.46, −1.74); *P* = 0.0001]. In comparing acupuncture with WM, six studies met the inclusion criteria, and acupuncture outperformed WM in reducing the DLQI [MD = −3.43 (−5.35, −1.51); *P* = 0.0005]. Four studies comparing acupuncture with WC were included. Acupuncture outperformed WC in reducing the DLQI [MD = −5.04 (−6.52, −3.55); *P* < 0.00001]. Based on differences in the intervention groups, we conducted a subgroup analysis (Supplementary Figure S4) on the intervention methods. The results indicated that MA [MD = −3.90 (−5.03, −2.77); *P* < 0.00001] and warm moxibustion [MD = −3.97 (−8.01, 0.08); *P* = 0.05], significantly reduced the DLQI compared with the controls. However, no significant difference was observed among the three groups (*P* = 0.97).

#### 3.3.3 Changes in serum IgE levels

Seven studies reported the serum IgE levels before and after treatment, with an overall sample size of 680 cases (Supplementary Figure S8). Using a random-effects model for analysis, the results indicated that the acupuncture and the control groups exhibited reduced serum IgE levels; however, the difference between the two groups was statistically non-significant (*P* = 0.14). In the TSA, the Z-curve crossed the conventional test boundary and the effectiveness boundary but did not reach the RIS boundary, indicating that the results of this meta-analysis had certain robustness; nevertheless, a larger sample size is still needed to confirm this conclusion (Supplementary Figure S9). Egger's test revealed no significant publication bias (Supplementary Figure S10). The GRADE tool rated the quality of the evidence as moderate (Figure 5).

#### 3.3.4 Safety evaluation

Among the included trials, 11 reported AEs. The main adverse effects reported in these studies included hematoma, pain, vesicle, giddiness and headache, nausea, and erythema. Regarding adverse effect incidence rates, the acupuncture group (2.76%; 6/217 participants) exhibited a statistically non-significant difference compared with the WM group (5.12%; 11/215 participants); however, it was significantly higher than the SA (2.95%; 8/271 participants) and the WC groups, which reported no adverse effects (Table 2).

**Table 2:** Safety evaluation.

**Intervention vs Control**	**Study**	**Intervention**			**Control**			**Occurrence rate (%)**	**Risk ratio (95% CI)**
		**Events**	**Adverse events reported**	**Total**	**Events**	**Adverse events reported**	**Total**		
MA vs WM	Anqi Dai 2014	2	Vesicle	30	1	–	30	6.50%	2.00 [0.19, 20.90]
	Haiyan Gu 2020	1	Giddy	45	1	Nausea	45	5.50%	1.00 [0.06, 15.50]
	Jing Liang 2013	0	–	35	1	Drowsiness/giddyheadache	35	4.60%	0.33 [0.01, 7.91]
	Liangnan Zhang 2019	1	Giddy	45	1	Nausea	45	5.50%	1.00 [0.06, 15.50]
	Lu Cao 2024	2	Hematoma	31	4	Giddy nausea	29	8.90%	0.47 [0.09, 2.36]
	Wenna Shu 2023	0	Headache/xerostomia	31	3	–	31	5.10%	0.14 [0.01, 2.66]
	Total events	6		217	11		215	41.60%	0.62 [0.23, 1.65]
MA vs SA	Hui Zheng 2023 A	7	Hematoma /Sharp pain lasting >1 h	55	0	–	110	5.20%	29.73 [1.73, 511.25]
	Leixiao Zhang 2021 A	5	Hematoma/pain	9	0	–	18	5.40%	20.90 [1.28, 340.94]
	Shengyuan Qu 2023	4	Hematoma/pain	37	1	Hematoma	37	7.10%	4.00 [0.47, 34.11]
	Xianjun Xiao 2021 A	6	Hematoma	33	0	–	67	5.20%	26.00 [1.51, 448.06]
	Yunzhou Shi 2023	13	Slight hematoma	41	7	Slight hematoma /sharp pain	39	11.80%	1.77 [0.79, 3.96]
	Total events	35		175	8		271	34.80%	7.18 [1.67, 30.90]
MA vs WC	Hui Zheng 2023 B	8	Hematoma /Sharp pain lasting >1 h	55	0	–	110	5.30%	33.70 [1.98, 573.30]
	Leixiao Zhang 2021 B	5	Hematoma/pain	9	0	–	17	5.40%	19.80 [1.22, 322.35]
	Xianjun Xiao 2021 B	7	Hematoma	34	0	–	68	5.30%	29.57 [1.74, 502.94]
	Total events	20		98	0		195	15.90%	26.93 [5.29, 137.13]
MA vs CON	Total (95% CI)	61		490	19		681	100.00%	3.31 [1.32, 8.29]

### 3.4 Quality of evidence

Employing the GRADE methodology, we evaluated the evidence quality from 18 RCTs across three outcomes: UAS7, DLQI, and IgE level changes. The evidence quality was moderate for UAS7 reduction, low for DLQI reduction, and moderate for IgE level changes, with no outcomes achieving high-quality evidence. The primary contributors to the downgrade in evidence quality were significant study heterogeneity and suspected publication bias (Figure 5).

## 4 Discussion

Pruritus and wheals associated with CU can severely impact patient's daily life and sleep quality, potentially affecting their mood and mental health. However, the etiology is often unclear, and treatment can be complex, necessitating long-term management and adequate symptomatic therapy (17, 18). Acupuncture is an alternative preventive treatment for difficult-to-control urticaria episodes (18). This review included 18 studies with 1,829 participants, comparing the acupuncture group with the control groups, which included WM, SA, and WC. The meta-analysis, assessing UAS7, DLQI, and IgE changes, indicates that acupuncture effectively alleviates symptoms, improves disease activity in patients with urticaria, and improves patients' quality of life, affirming its efficacy and safety.

### 4.1 Summary of main findings

The UAS7 is a valuable clinical instrument for assessing the severity of conditions and therapeutic outcomes in patients with CU (49). Compared with an average reduction of 4.29 points in the UAS7 following SA, acupuncture treatment resulted in a 10.19-point decrease. In contrast to the average decline of 1.51 points in the UAS7 of the WC group, acupuncture treatment resulted in a 10.18-point decrease. Additionally, TSA indicated sufficient information size, and the quality of evidence was moderate. Our study, alongside several previous systematic reviews, indicated that acupuncture is effective in alleviating CU attacks (14, 15, 50) and significantly improves UAS7 scores over SA and WC (20, 33) despite not reaching the MCID threshold (51, 52), highlighting a gap between statistical significance and clinical relevance. This suggests that while the reduction is statistically significant, its clinical meaningfulness requires further investigation, echoing Yunzhou Shi and Hui Zheng's findings.

The DLQI is designed to assess the quality of life of patients with skin diseases (53). The SA exhibited a 2.44-point decrease, whereas acupuncture treatment resulted in a 5.97-point decrease. Compared with the WM group, which exhibited a 7.09-point reduction in the DLQI after treatment, the acupuncture group exhibited a more significant decrease of 10.15 points. Compared with the WC group, which had a 0.01-point increase in the DLQI after treatment, the acupuncture group exhibited a 6.07-point reduction. Acupuncture treatment improves the quality of life of patients with CU more than positive drug therapy and SA, with statistically significant differences. Subgroup analysis based on acupuncture techniques (manual acupuncture and warm moxibustion) revealed no significant difference between them (*P* = 0.97). TSA indicated sufficient information; however, the quality of the evidence was low. Previous RCT (20) revealed that acupuncture treatment for urticaria results in a decrease in DLQI over time, exhibiting better outcomes compared with SA and WC.

IgE, linked to allergic reactions, may reflect urticaria severity (53). Acupuncture for CU treatment may exert its effects by modulating the immune response, particularly IgE levels (16). Acupuncture, like WM, can reduce serum IgE levels, with statistically non-significant differences. TSA indicated insufficient information, and the quality of the evidence was rated as moderate, suggesting the need for higher-quality RCTs in the future.

Regarding safety and adverse effect incidence rates, the WC exhibited no adverse effects, and no significant difference was observed between MA and WM. However, compared with SA, acupuncture showed a higher incidence of ecchymosis and pain, possibly due to stricter standards for real acupuncture versus the more lenient SA, with some SA not penetrating the skin.

### 4.2 Strengths and limitations

To our knowledge, this study is the first to employ a meta-analysis with TSA to validate the efficacy of acupuncture in treating chronic urticaria, using the GRADE system to assess evidence quality. Our comprehensive search strategy, rigorous risk of bias assessment, and detailed safety evaluation contribute to the reliability of our findings, potentially informing clinical practices and health policies. However, this study has certain limitations. First, in the TSA, strict control of false-positive errors increased the possibility of false-negative risks, resulting in the TSA results tending to be conservative. Second, the review was limited to Chinese and English literature, excluding other languages. Third, varied sample sizes and low evidence quality introduced uncertainty. In addition, the duration of acupuncture treatment, frequency, and acupoint selection were rarely mentioned or discussed in the studies. Finally, the choice of WM for the control group was inadequately investigated.

### 4.3 Implications for future research

Future research should prioritize several key areas to strengthen the evidence base for acupuncture in CU. First, the long-term therapeutic effect of acupuncture on CU warrants formal investigation through future studies designed with longer-term assessments (3–6 months) to confirm its durability. The current paucity of high-quality comparative trials between acupuncture and antihistamines, the standard treatment, indicates large-scale, pragmatic RCTs that directly compare acupuncture with first-line antihistamines are essential to definitively establish its relative efficacy and clinical role. The efficacy variations observed among different acupuncture techniques also highlight the importance of head-to-head comparative studies to identify optimal modalities, alongside standardized protocol reporting per STRICTA guidelines. Finally, correlating clinical improvement with biomarker changes will be crucial for elucidating mechanisms of action and identifying predictors of treatment response.

## 5 Conclusion

Our study demonstrated that acupuncture can alleviate skin symptoms in patients with urticaria and improve their quality of life. However, to better integrate acupuncture into clinical practice, more high-quality, large-sample, and standardized studies are needed to determine its efficacy, optimal treatment parameters, and long-term effects.

## Data Availability

The raw data supporting the conclusions of this article will be made available by the authors, without undue reservation.

## Supplementary material

Supplementary File S1 Search strategies of each database (DOCX).

Supplementary File S2 STRICTA (XLSX).

Supplementary File S3 Parameter Settings for Sequential analysis of trials (DOCX).

Supplementary Figure S1 Forest plot showing the comparison of UAS7 scores between the acupuncture group and the control group during the follow-up period.

Supplementary Figure S2 Egger's test for UAS7.

Supplementary Figure S3 Forest plot showing the comparison of DLQI scores between the acupuncture group and control group.

Supplementary Figure S4 Forest plot showing the comparison of DLQI scores between the acupuncture subgroups and the control group.

Supplementary Figure S5 Forest plot showing the comparison of DLQI scores between the acupuncture group and the control group during the follow-up period.

Supplementary Figure S6 Trial sequential analysis of DLQI adjusted boundaries.

Supplementary Figure S7 Egger's test for DLQI.

Supplementary Figure S8 Forest plot showing the comparison of serum IgE level between the acupuncture group and the control group.

Supplementary Figure S9 Trial sequential analysis of IgE adjusted boundaries.

Supplementary Figure S10 Egger's test for IgE.
