# Supplementary material for: Acupuncture for chronic urticaria: a systematic review and meta-analysis with trial sequential analysis
Source: Front Neurol. 2026 Jan 21;16:1650418. doi: 10.3389/fneur.2025.1650418 (PMC12867926; doi:10.3389/fneur.2025.1650418)
Supplement: Supplementary File S1 — Search strategies of each database (DOCX). [file Supplementary_file_1.docx]

**2025.9.30**

**PubMed--59**

#1 "urticaria"[MeSH Terms] OR "angioedema"[MeSH Terms]

#2 "chronic urticaria["[Title/Abstract] OR "chronic idiopathic urticaria"[Title/Abstract] OR "chronic spontaneous urticaria"[Title/Abstract] OR "hives"[Title/Abstract] OR "nettle rash"[Title/Abstract] OR "weals"[Title/Abstract] OR "wheals"[Title/Abstract] OR "urticaria"[Title/Abstract] OR " urticarial vasculitis "[Title/Abstract]

#3 #1 OR #2

#4 "Acupuncture therapy"[MeSH Terms]

#5 "acupuncture therapy"[Title/Abstract] OR "acupuncture"[Title/Abstract] OR "acupuncture-moxibustion"[Title/Abstract] OR "meridian*"[Title/Abstract] OR "acupoint*"[Title/Abstract] OR "warm needling"[Title/Abstract] OR "warm acupuncture"[Title/Abstract] OR "acupuncture plus moxibustion"[Title/Abstract] OR "electronic acupuncture"[Title/Abstract] OR "electro-acupuncture"[Title/Abstract] OR "electroacupuncture"[Title/Abstract] OR "fire acupuncture"[Title/Abstract] OR "needle"[Title/Abstract] OR "body acupuncture"[Title/Abstract] OR "manual-acupuncture"[Title/Abstract] OR "auricular needle"[Title/Abstract] OR "ear acupuncture"[Title/Abstract] OR "moxibustion["[Title/Abstract] OR "wrist-ankle acupuncture"[Title/Abstract] OR "scalp acupuncture"[Title/Abstract]

#6 #4 OR #5

#7 "Randomized Controlled Trials as Topic"[MeSH Terms] OR "random allocation"[MeSH Terms] OR "randomized controlled trial"[Publication Type] OR "controlled clinical trial"[Publication Type] OR "clinical trial"[Publication Type] OR "clinical study"[Title/Abstract] OR "trial"[Title/Abstract] OR "placebo"[Title/Abstract] OR "random*"[Title/Abstract]

#8 #3 AND #6 AND #7

**EMBASE--116**

#1 ‘urticaria’/exp/mj OR ‘angioedema’/exp/mj

#2 ‘chronic urticaria’:ti,ab,kw OR ‘chronic idiopathic urticaria’:ti,ab,kw OR ‘chronic spontaneous urticaria’:ti,ab,kw OR ‘hives’:ti,ab,kw OR ‘nettle rash’:ti,ab,kw OR ‘weals’:ti,ab,kw OR ‘wheals’:ti,ab,kw OR ‘urticaria’:ti,ab,kw OR ‘urticarial vasculitis’:ti,ab,kw

#3 #1 OR #2

#4 ‘Acupuncture therapy’/exp/mj

#5 ‘acupuncture therapy’:ti,ab,kw OR ‘acupuncture’:ti,ab,kw OR ‘acupuncture-moxibustion’:ti,ab,kw OR ‘meridian*’:ti,ab,kw OR ‘acupoint*’:ti,ab,kw OR ‘warm needling’:ti,ab,kw OR ‘warm acupuncture’:ti,ab,kw OR ‘acupuncture plus moxibustion’:ti,ab,kw OR ‘electronic acupuncture’:ti,ab,kw OR ‘electro-acupuncture’:ti,ab,kw OR ‘electroacupuncture’:ti,ab,kw OR ‘fire acupuncture’:ti,ab,kw OR ‘needle’:ti,ab,kw OR ‘body acupuncture’:ti,ab,kw OR ‘manual-acupuncture’:ti,ab,kw OR ‘auricular needle’:ti,ab,kw OR ‘ear acupuncture’:ti,ab,kw OR ‘moxibustion’:ti,ab,kw OR ‘wrist-ankle acupuncture’:ti,ab,kw OR ‘scalp acupuncture’:ti,ab,kw

#6 #4 OR #5

#7 ‘randomized controlled trials as topic’/exp/mj OR ‘randomized controlled trial’/exp/mj OR ‘clinical’:ti,ab,kw OR ‘trial’:ti,ab,kw OR ‘clinical trial’:ti,ab,kw OR ‘random’:ti,ab,kw OR '**placebo**':ti,ab,kw

#8 #3 AND #6 AND #7

**Cochrane Library—75**

#1 MeSH descriptor: [Urticaria] explode all trees 1039

#2 MeSH descriptor: [Angioedema] explode all trees 293

#3 chronic urticaria:ti,ab,kw OR chronic idiopathic urticaria:ti,ab,kw OR chronic spontaneous urticaria:ti,ab,kw OR hives:ti,ab,kw OR nettle rash:ti,ab,kw OR weals:ti,ab,kw OR wheals:ti,ab,kw OR urticaria:ti,ab,kw OR urticarial vasculitis:ti,ab,kw 3700

#4 #1 OR #2 OR #3 3948

#5 MeSH descriptor: [Acupuncture Therapy] explode all trees 6992

#6 acupuncture therapy:ti,ab,kw OR acupuncture:ti,ab,kw OR acupuncture-moxibustion:ti,ab,kw OR meridian*:ti,ab,kw OR acupoint*:ti,ab,kw OR warm needling:ti,ab,kw OR warm acupuncture:ti,ab,kw OR acupuncture plus moxibustion:ti,ab,kw OR electronic acupuncture:ti,ab,kw OR electro-acupuncture:ti,ab,kw OR electroacupuncture:ti,ab,kw OR fire acupuncture:ti,ab,kw OR needle:ti,ab,kw OR body acupuncture:ti,ab,kw OR manual-acupuncture:ti,ab,kw OR auricular needle:ti,ab,kw OR ear acupuncture:ti,ab,kw OR moxibustion:ti,ab,kw OR wrist-ankle acupuncture’:ti,ab,kw OR ‘scalp acupuncture’:ti,ab,kw 40780

#7 #5 OR #6 440780

#8 MeSH descriptor: [Randomized Controlled Trials as Topic] explode all trees 54784

#9 MeSH descriptor: [Randomized Controlled Trial] explode all trees 37

#10 clinical:ti,ab,kw OR trial:ti,ab,kw OR clinical trial:ti,ab,kw OR random:ti,ab,kw OR 'placebo':ti,ab,kw 1444232

#11 #8 OR #9 OR #10 1446682

#12 #4 AND #7 AND #11 65

**WOS--58**

TS=(‘urticaria’ OR ‘angioedema’ OR ‘chronic urticaria’ OR ‘chronic idiopathic urticaria’ OR ‘chronic spontaneous urticaria’ OR ‘hives’ OR ‘nettle rash’ OR ‘weals’ OR ‘wheals’ OR ‘urticaria’ OR ‘urticarial vasculitis’) AND TS=(‘acupuncture therapy’ OR ‘acupuncture’ OR ‘acupuncture-moxibustion’ OR ‘meridian*’ OR ‘acupoint*’ OR ‘warm needling’ OR ‘warm acupuncture’ OR ‘acupuncture plus moxibustion’ OR ‘electronic acupuncture’ OR ‘electro-acupuncture’ OR ‘electroacupuncture’ OR ‘fire acupuncture’ OR ‘needle’ OR ‘body acupuncture’ OR ‘manual-acupuncture’ OR ‘auricular needle’ OR ‘ear acupuncture’ OR ‘moxibustion’ OR ‘wrist-ankle acupuncture’ OR ‘scalp acupuncture’) AND TS=(‘randomized controlled trials as topic’ OR ‘randomized controlled trial’ OR ‘clinical’ OR ‘trial’ OR ‘clinical trial’ OR ‘random’ OR ‘**placebo**’)

AMED--23

S1 SU urticaria OR angioedema OR chronic urticaria OR chronic idiopathic urticaria OR chronic spontaneous urticaria OR hives OR nettle rash OR weals OR wheals OR urticaria OR urticarial vasculitis

S2 SU acupuncture therapy OR acupuncture OR acupuncture-moxibustion OR meridian* OR acupoint* OR warm needling OR warm acupuncture OR acupuncture plus moxibustion OR electronic acupuncture OR electro-acupuncture OR electroacupuncture OR fire acupuncture OR needle OR body acupuncture OR manual-acupuncture OR auricular needle OR ear acupuncture OR moxibustion OR wrist-ankle acupuncture OR scalp acupuncture

S3 SU randomized controlled trials as topic OR randomized controlled trial OR clinical OR trial OR clinical trial OR random OR **placebo**

S4 S1 AND S2 AND S3

CINAHL--29

S1 SU urticaria OR angioedema OR chronic urticaria OR chronic idiopathic urticaria OR chronic spontaneous urticaria OR hives OR nettle rash OR weals OR wheals OR urticaria OR urticarial vasculitis

S2 SU acupuncture therapy OR acupuncture OR acupuncture-moxibustion OR meridian* OR acupoint* OR warm needling OR warm acupuncture OR acupuncture plus moxibustion OR electronic acupuncture OR electro-acupuncture OR electroacupuncture OR fire acupuncture OR needle OR body acupuncture OR manual-acupuncture OR auricular needle OR ear acupuncture OR moxibustion OR wrist-ankle acupuncture OR scalp acupuncture

S3SU randomized controlled trials as topic OR randomized controlled trial OR clinical OR trial OR clinical trial OR random OR **placebo**

S4 S1 AND S2 AND S3

**CNKI****--580**

(TKA=(‘荨麻疹’+‘血管性水肿’+‘血管神经水肿’+‘风疹’+‘风团’+‘瘾疹’) OR SU=(‘荨麻疹’+‘血管性水肿’)) AND (TKA=(‘针刺’+‘针灸’+‘体针’+‘手针’+‘电针’+‘温针’+‘毫针’+‘穴’+‘经络’+‘刃针’+‘火针’+‘针刀’+‘腹针’+‘浮针’+‘腕踝针’+‘干针’+‘眼针’+‘头针’+‘耳针’+‘灸’) OR SU=(‘针刺疗法’+‘针刺’+‘针灸疗法’)) AND (TKA=(‘随机’+‘对照’+‘临床疗效’) OR SU=(‘随机对照试验’))

**WF--247**

(主题:(荨麻疹 OR 血管性水肿) or 题名或关键词:(荨麻疹 OR 血管性水肿 OR 血管神经水肿 OR 风疹 OR 风团 OR 瘾疹)) and (主题:(针刺疗法 OR 针刺 OR 针灸疗法) or 题名或关键词:(针刺 OR 针灸 OR 体针 OR 手针 OR 电针 OR 温针 OR 毫针 OR 穴 OR 经络 OR 刃针 OR 火针 OR 针刀 OR 腹针 OR 浮针 OR 腕踝针 OR 干针 OR 眼针 OR 头针 OR 耳针OR 灸) ) and (主题:(随机对照试验) or 题名或关键词:(随机OR对照 OR 临床疗效))

**Chongqing VIP--34**

M=(荨麻疹 OR 血管性水肿 OR 血管神经水肿 OR 风疹 OR 风团 OR 瘾疹) and M=(针刺 OR 针灸 OR 体针 OR 手针 OR 电针 OR 温针 OR 毫针 OR 穴 OR 经络 OR 刃针 OR 火针 OR 针刀 OR 腹针 OR 浮针 OR 腕踝针 OR 干针 OR 眼针 OR 头针 OR 耳针OR 灸) and M=(随机 OR 对照 OR 临床疗效)

**CBM--661**

1. "荨麻疹"[加权:扩展] OR "血管性水肿"[加权:扩展]
2. “荨麻疹”[常用字段:智能] OR “血管性水肿”[常用字段:智能] OR “血管神经水肿”[常用字段:智能] OR “风疹”[常用字段:智能] OR “风团”[常用字段:智能] OR “瘾疹”[常用字段:智能]
3. 1 OR 2
4. "针刺疗法"[加权:扩展] OR "针刺"[加权:扩展] OR "针灸疗法"[加权:扩展]
5. “针刺”[常用字段:智能] OR “针灸”[常用字段:智能] OR “体针”[常用字段:智能] OR “手针”[常用字段:智能] OR “电针”[常用字段:智能] OR “温针”[常用字段:智能] OR “毫针”[常用字段:智能] OR “穴”[常用字段:智能] OR “经络”[常用字段:智能] OR “刃针”[常用字段:智能] OR “火针”[常用字段:智能] OR “针刀”[常用字段:智能] OR “腹针”[常用字段:智能] OR “浮针”[常用字段:智能] OR “腕踝针”[常用字段:智能] OR “干针”[常用字段:智能] OR “眼针”[常用字段:智能] OR “头针”[常用字段:智能] OR “耳针”[常用字段:智能] OR “灸”[常用字段:智能]

6 4 OR 5

7 "随机对照试验"[不加权:扩展]

8 "随机"[常用字段:智能] OR "对照"[常用字段:智能] OR "临床疗效"[常用字段:智能]

9 7 OR 8

10 3 AND 6 AND 9

(("随机"[常用字段:智能] OR "对照"[常用字段:智能] OR "临床疗效"[常用字段:智能]) OR ("随机对照试验"[不加权:扩展])) AND (((“针刺”[常用字段:智能] OR “针灸”[常用字段:智能] OR “体针”[常用字段:智能] OR “手针”[常用字段:智能] OR “电针”[常用字段:智能] OR “温针”[常用字段:智能] OR “毫针”[常用字段:智能] OR “穴”[常用字段:智能] OR “经络”[常用字段:智能] OR “刃针”[常用字段:智能] OR “火针”[常用字段:智能] OR “针刀”[常用字段:智能] OR “腹针”[常用字段:智能] OR “浮针”[常用字段:智能] OR “腕踝针”[常用字段:智能] OR “干针”[常用字段:智能] OR “眼针”[常用字段:智能] OR “头针”[常用字段:智能] OR “耳针”[常用字段:智能] OR “灸”[常用字段:智能]) OR ("针刺疗法"[加权:扩展] OR "针刺"[加权:扩展] OR "针灸疗法"[加权:扩展])) AND ((“荨麻疹”[常用字段:智能] OR “血管性水肿”[常用字段:智能] OR “血管神经水肿”[常用字段:智能] OR “风疹”[常用字段:智能] OR “风团”[常用字段:智能] OR “瘾疹”[常用字段:智能]) OR ("荨麻疹"[加权:扩展] OR "血管性水肿"[加权:扩展])))
