# Supplementary material for: Acupuncture for chronic urticaria: a systematic review and meta-analysis with trial sequential analysis
Source: Front Neurol. 2026 Jan 21;16:1650418. doi: 10.3389/fneur.2025.1650418 (PMC12867926; doi:10.3389/fneur.2025.1650418)
Supplement: Supplementary File S3 — Parameter Settings for Sequential analysis of trials (DOCX). [file Supplementary_file_3.docx]

Parameter Settings for Sequential analysis of trials

①Conventional Test Boundary: Boundary Type was set to Two-sided, with Type 1 Error set to 5%.

②Alpha-Spending Boundaries: Boundary Type was set to Two-sided.Information Axis was set to Sample Size. α-spending function was set to O'Brien-Fleming. Information Size was set to Estimate. Power was set to 95%. Mean Difference and Variance were both set to Empirical. Heterogeneity Correction was set to Model Variance Based.

③Law of the Iterated Logarithm: Boundary Type was set to Two-sided. Type 1 Error was set to 5%. λ (penalty) was set to 2.0.
