# Supplementary material for: Acupuncture for chronic urticaria: a systematic review and meta-analysis with trial sequential analysis
Source: Front Neurol. 2026 Jan 21;16:1650418. doi: 10.3389/fneur.2025.1650418 (PMC12867926; doi:10.3389/fneur.2025.1650418)
Supplement: Supplementary Figure S1 — Forest plot showing the comparison of UAS7 scores between the acupuncture group and the control group during the follow-up period. [file Supplementary_file_4.pdf]

	MA			CON				Mean Difference
Study or Subgroup	Mean	SD	Total	Mean	SD	Total	Weight	IV, Random, 95% CI
Lu Wang 2021 B	-12.83	1.38	12	-1.08	2.05	23	13.4%	-11.75 [-12.90, -10.60]
Xianjun Xiao 2021 B	-12.51	9.9	34	-4.38	8.06	68	10.7%	-8.13 [-11.97, -4.29]
Shengyuan Qu 2023	-13.66	7.47	37	-6.5	9.36	37	10.7%	-7.16 [-11.02, -3.30]
Lu Wang 2021 A	-12.83	1.38	12	-5.68	1.81	25	13.4%	-7.15 [-8.21, -6.09]
Hui Zheng 2023 B	-11.4	8.83	55	-4.9	9.1	110	11.8%	-6.50 [-9.39, -3.61]
Xianjun Xiao 2021 A	-12.51	9.9	33	-6.19	8.79	67	10.5%	-6.32 [-10.30, -2.34]
Hui Zheng 2023 A	-11.4	8.83	55	-6.2	9.36	110	11.8%	-5.20 [-8.12, -2.28]
Yunzhou Shi 2023	-11.17	11.42	41	-9.56	11.4	39	9.3%	-1.61 [-6.61, 3.39]
Lu Cao 2024	-4	11.4	31	-13	11.55	29	8.4%	9.00 [3.19, 14.81]
**Total (95% CI)**			**310**			**508**	**100.0%**	**-5.61 [-8.30, -2.92]**

Heterogeneity: Tau² = 13.75; Chi² = 87.46, df = 8 (P < 0.00001); I² = 91%

Test for overall effect: Z = 4.09 (P < 0.0001)

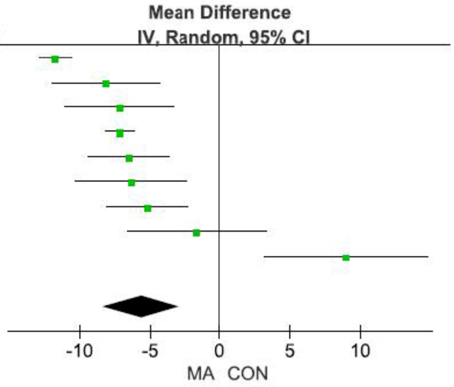

FIGURE S1

Forest plot showing the comparison of UAS7 scores between the acupuncture group and the control group during the follow-up period

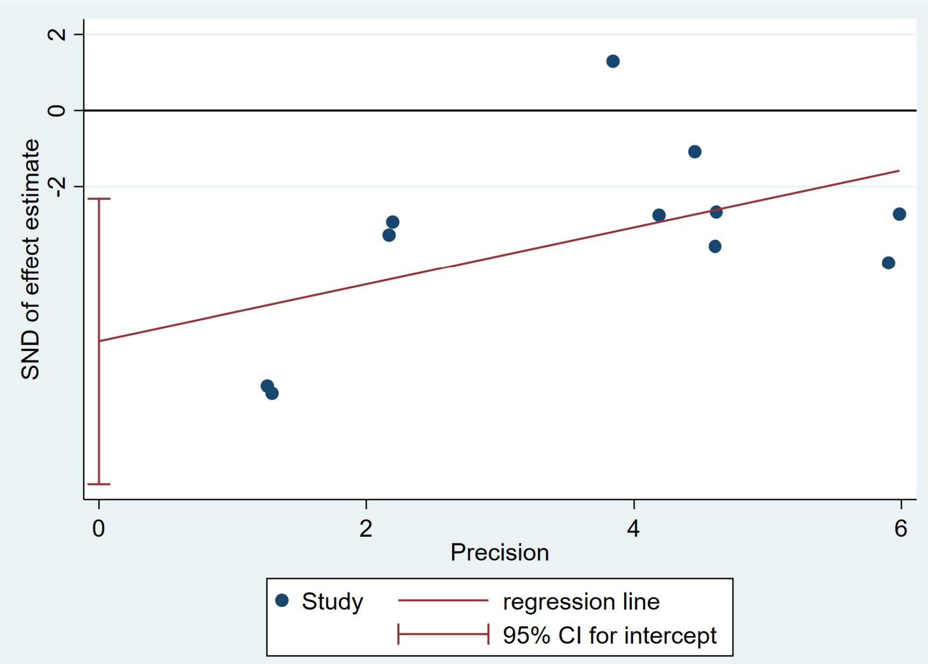

FIGURE S2
Egger's test for UAS7

Study or Subgroup	MA Mean	MA SD	MA Total	CON Mean	CON SD	CON Total	Weight	Mean Difference IV, Random, 95% CI
**4.2.1 MA vs SA**								
Hui Zheng 2023 A	-5.1	5.89	55	-2.3	5.62	110	7.0%	-2.80 [-4.68, -0.92]
Leixiao Zhang 2021 A	-7.5	6.11	9	-3	5.61	18	3.2%	-4.50 [-9.26, 0.26]
Lu Wang 2021 A	-5.79	1.07	12	-0.44	0.84	25	8.6%	-5.35 [-6.04, -4.66]
Xianjun Xiao 2021 A	-5.87	6.83	33	-1.58	3.68	67	6.0%	-4.29 [-6.78, -1.80]
Yunzhou Shi 2023	-5.61	5.96	41	-4.9	5.95	39	5.8%	-0.71 [-3.32, 1.90]
**Subtotal (95% CI)**			**150**			**259**	**30.5%**	**-3.60 [-5.46, -1.74]**
Heterogeneity: Tau² = 3.00; Chi² = 16.39, df = 4 (P = 0.003); I² = 76%								
Test for overall effect: Z = 3.80 (P = 0.0001)								
**4.2.2 MA vs WM**								
Anqi Dai 2014	-5.44	2.8	30	-3.57	2.47	30	7.8%	-1.87 [-3.21, -0.53]
Jing Liang 2013	-13	4.77	35	-10.2	6.09	35	5.8%	-2.80 [-5.36, -0.24]
Nan Ge 2024	-6.49	1.74	31	-4.71	1.67	31	8.5%	-1.78 [-2.63, -0.93]
Wenna Shu 2023	-13.9	1.76	31	-7.9	1.73	31	8.4%	-6.00 [-6.87, -5.13]
Wenyin Wu 2020	-17.47	5.14	53	-10.91	4.76	53	7.0%	-6.56 [-8.45, -4.67]
Zhongxun Wang 2021	-5.59	2.65	34	-3.9	2.07	31	8.1%	-1.69 [-2.84, -0.54]
**Subtotal (95% CI)**			**214**			**211**	**45.6%**	**-3.43 [-5.35, -1.51]**
Heterogeneity: Tau² = 5.18; Chi² = 72.89, df = 5 (P < 0.00001); I² = 93%								
Test for overall effect: Z = 3.50 (P = 0.0005)								
**4.2.3 MA vs WC**								
Hui Zheng 2023 B	-5.1	5.87	55	-1.2	5.62	110	7.0%	-3.90 [-5.77, -2.03]
Leixiao Zhang 2021 B	-7.5	6.11	9	3.26	6.59	17	2.9%	-10.76 [-15.83, -5.69]
Lu Wang 2021 B	-5.79	1.07	12	-0.83	1.03	23	8.6%	-4.96 [-5.70, -4.22]
Xianjun Xiao 2021 B	-5.87	6.83	34	-1.18	6.77	68	5.5%	-4.69 [-7.49, -1.89]
**Subtotal (95% CI)**			**110**			**218**	**23.9%**	**-5.04 [-6.52, -3.55]**
Heterogeneity: Tau² = 1.10; Chi² = 6.26, df = 3 (P = 0.10); I² = 52%								
Test for overall effect: Z = 6.65 (P < 0.00001)								
**Total (95% CI)**			**474**			**688**	**100.0%**	**-3.92 [-4.97, -2.87]**
Heterogeneity: Tau² = 3.23; Chi² = 115.13, df = 14 (P < 0.00001); I² = 88%								
Test for overall effect: Z = 7.30 (P < 0.00001)								
Test for subgroup differences: Chi² = 2.23, df = 2 (P = 0.33), I² = 10.5%								

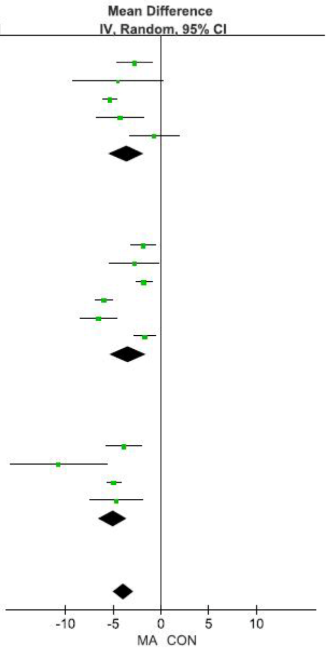

FIGURE S3

Forest plot showing the comparison of DLQI scores between acupuncture group and control group

Study or Subgroup	MA Mean	MA SD	MA Total	CON Mean	CON SD	CON Total	Weight	Mean Difference IV, Random, 95% CI
**4.3.1 Manual acupuncture**								
Hui Zheng 2023 A	-5.1	5.89	55	-2.3	5.62	110	7.0%	-2.80 [-4.68, -0.92]
Hui Zheng 2023 B	-5.1	5.89	55	-1.2	5.62	110	7.0%	-3.90 [-5.78, -2.02]
Jing Liang 2013	-13	4.77	35	-10.2	6.09	35	5.8%	-2.80 [-5.36, -0.24]
Leixiao Zhang 2021 A	-7.5	6.11	9	-3	5.61	18	3.2%	-4.50 [-9.26, 0.26]
Leixiao Zhang 2021 B	-7.5	6.11	9	3.26	6.59	17	2.9%	-10.76 [-15.83, -5.69]
Lu Wang 2021 A	-5.79	1.07	12	-0.44	0.84	25	8.6%	-5.35 [-6.04, -4.66]
Lu Wang 2021 B	-5.79	1.07	12	-0.83	1.03	23	8.6%	-4.96 [-5.70, -4.22]
Nan Ge 2024	-6.49	1.74	31	-4.71	1.67	31	8.5%	-1.78 [-2.63, -0.93]
Wenyin Wu 2020	-17.47	5.14	53	-10.91	4.76	53	7.0%	-6.56 [-8.45, -4.67]
Xianjun Xiao 2021 A	-5.87	6.83	33	-1.58	3.68	67	6.0%	-4.29 [-6.78, -1.80]
Xianjun Xiao 2021 B	-5.87	6.83	34	-1.18	6.77	68	5.5%	-4.69 [-7.49, -1.89]
Yunzhou Shi 2023	-5.61	5.96	41	-4.9	5.95	39	5.8%	-0.71 [-3.32, 1.90]
Zhongxun Wang 2021	-5.59	2.65	34	-3.9	2.07	31	8.1%	-1.69 [-2.84, -0.54]
**Subtotal (95% CI)**			**413**			**627**	**83.7%**	**-3.90 [-5.03, -2.77]**
Heterogeneity: Tau² = 3.09; Chi² = 85.74, df = 12 (P < 0.00001); I² = 86%								
Test for overall effect: Z = 6.75 (P < 0.00001)								
**4.3.2 Warm moxibustion**								
Anqi Dai 2014	-5.44	2.8	30	-3.57	2.47	30	7.8%	-1.87 [-3.21, -0.53]
Wenna Shu 2023	-13.9	1.76	31	-7.9	1.73	31	8.4%	-6.00 [-6.87, -5.13]
**Subtotal (95% CI)**			**61**			**61**	**16.3%**	**-3.97 [-8.01, 0.08]**
Heterogeneity: Tau² = 8.20; Chi² = 25.80, df = 1 (P < 0.00001); I² = 96%								
Test for overall effect: Z = 1.92 (P = 0.05)								
**Total (95% CI)**			**474**			**688**	**100.0%**	**-3.92 [-4.97, -2.87]**
Heterogeneity: Tau² = 3.23; Chi² = 115.13, df = 14 (P < 0.00001); I² = 88%								
Test for overall effect: Z = 7.29 (P < 0.00001)								
Test for subgroup differences: Chi² = 0.00, df = 1 (P = 0.97), I² = 0%								

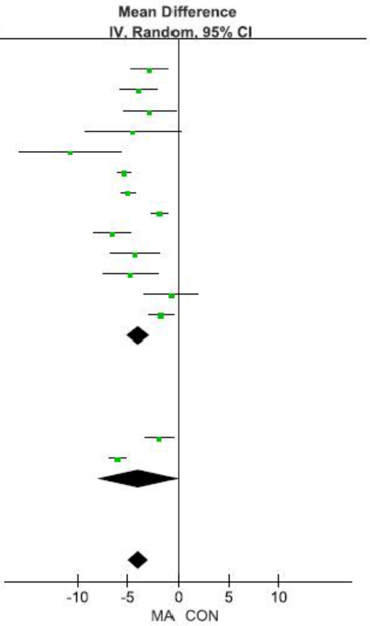

FIGURE S4
Forest plot showing the comparison of DLQI scores between the acupuncture subgroup and the control group

	MA			CON				Mean Difference	Mean Difference
Study or Subgroup	Mean	SD	Total	Mean	SD	Total	Weight	IV, Random, 95% CI	IV, Random, 95% CI
Hui Zheng 2023 A	-5.9	5.62	110	-3.6	5.62	110	14.4%	-2.30 [-3.79, -0.81]	
Hui Zheng 2023 B	-5.9	5.62	110	-3.7	5.62	110	14.4%	-2.20 [-3.69, -0.71]	
Lu Wang 2021 A	-6.66	1.07	24	-2	0.83	25	21.6%	-4.66 [-5.20, -4.12]	
Lu Wang 2021 B	-6.66	1.07	24	-2.13	1.05	23	21.1%	-4.53 [-5.14, -3.92]	
Xianjun Xiao 2021 A	-6.72	6.87	67	-3.69	4.2	67	11.4%	-3.03 [-4.96, -1.10]	
Xianjun Xiao 2021 B	-6.72	6.87	67	-3.69	5.01	68	10.8%	-3.03 [-5.06, -1.00]	
Yunzhou Shi 2023	-6.66	6.97	41	-5.67	6.97	39	6.4%	-0.99 [-4.05, 2.07]	
**Total (95% CI)**			**443**			**442**	**100.0%**	**-3.34 [-4.25, -2.44]**	

Heterogeneity: $Tau^2 = 0.92$; $Chi^2 = 24.11$, df = 6 (P = 0.0005); $I^2 = 75\%$

Test for overall effect: Z = 7.21 (P < 0.00001)

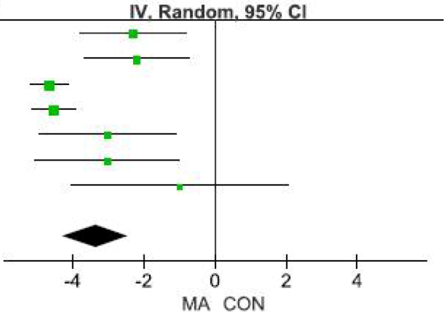

FIGURE S5

Forest plot showing the comparison of DLQI scores between the acupuncture group and the control group during the follow-up period

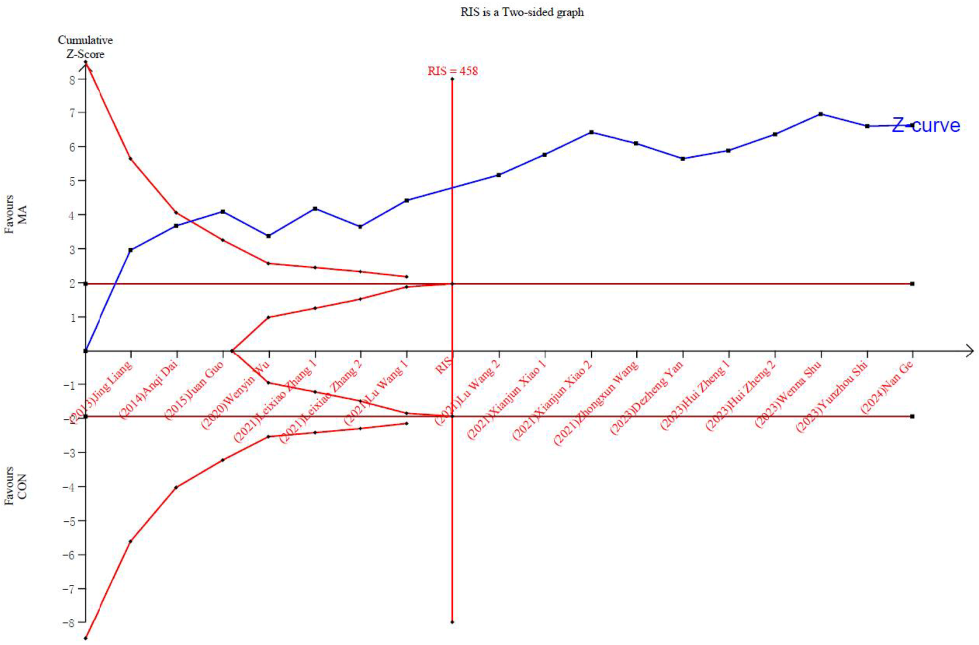

FIGURE S6
Trial sequential analysis of DLQI adjusted boundaries

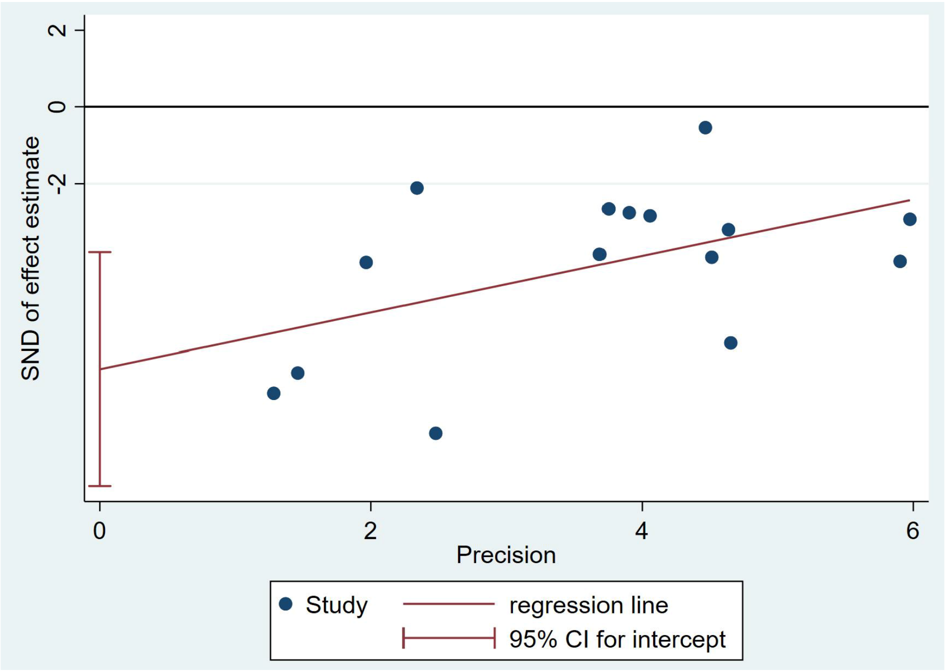

FIGURE S7
Egger's test for DLQI

Study or Subgroup	MA Mean	MA SD	MA Total	CON Mean	CON SD	CON Total	Weight	Mean Difference IV, Random, 95% CI
Haiyan Gu 2020	-82.68	14.39	44	-66.29	13.19	44	14.6%	-16.39 [-22.16, -10.62]
Hong Gao 2009	-28.93	88.19	30	-14.64	113.45	30	4.0%	-14.29 [-65.71, 37.13]
Liangnan Zhang 2019	-82.68	14.39	44	-66.29	13.19	44	14.6%	-16.39 [-22.16, -10.62]
Libin Gu A 2019	-83.61	13.19	78	-70.2	12.46	78	14.8%	-13.41 [-17.44, -9.38]
Libin Gu B 2019	-83.74	13.66	60	-70.41	13.02	60	14.7%	-13.33 [-18.10, -8.56]
Nan Ge 2024	-83.83	53.3	31	-73.13	66.06	31	7.8%	-10.70 [-40.58, 19.18]
Wenyin Wu 2020	-83.07	13.16	53	-70.42	12.72	53	14.7%	-12.65 [-17.58, -7.72]
Yurong Zheng 2021	-25.58	8.83	38	-45.73	8.5	37	14.8%	20.15 [16.23, 24.07]
**Total (95% CI)**			**378**			**377**	**100.0%**	**-9.01 [-21.10, 3.09]**

Heterogeneity: Tau² = 253.16; Chi² = 224.94, df = 7 (P < 0.00001); I² = 97%
Test for overall effect: Z = 1.46 (P = 0.14)

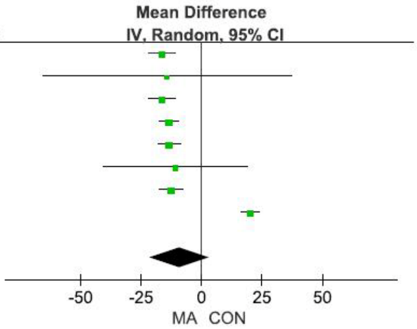

FIGURE S8
Forest plot showing the comparison of serum IgE level between the acupuncture group and the control group

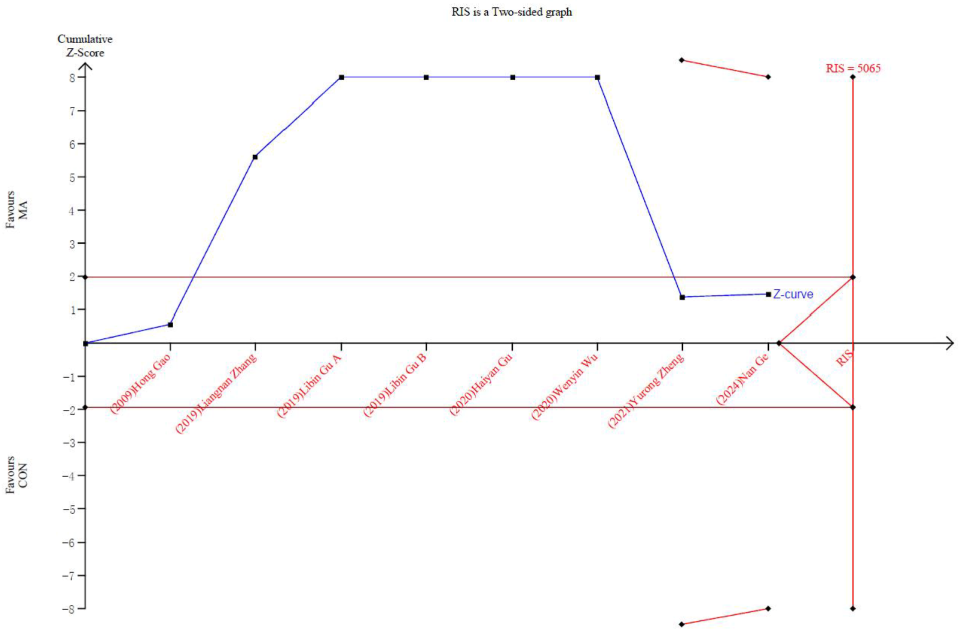

FIGURE S9
Trial sequential analysis of IgEadjusted boundaries

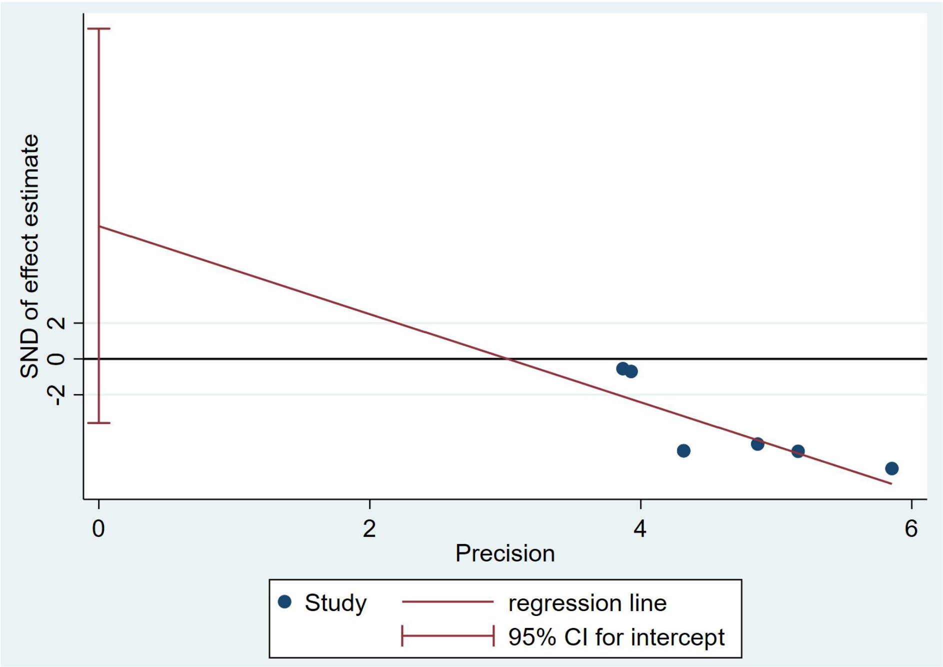

FIGURE S10
Egger's test for IgE
